# Emergence of the first XAFS/XRF beamline in the Middle East: providing studies of elements and their atomic/electronic structure in pluridisciplinary research fields

**DOI:** 10.1107/S1600577522005215

**Published:** 2022-05-26

**Authors:** Messaoud Harfouche, Mahmoud Abdellatief, Yazeed Momani, Anas Abbadi, Mohammad Al Najdawi, Mustafa Al Zoubi, Basil Aljamal, Salman Matalgah, Latif U. Khan, Andrea Lausi, Giorgio Paolucci

**Affiliations:** a SESAME (Synchrotron-light for Experimental Science and Applications in the Middle East), Allan 19252, Jordan; b Elettra-Sincrotrone Trieste SCpA, Strada Statale 14 – km 163,5 in AREA Science Park, Basovizza/Trieste 34149, Italy

**Keywords:** SESAME, synchrotron, XRF, XAS, XAFS, EXAFS, XANES, structure, optics, monochromator, catalysis, cultural heritage, materials

## Abstract

XAFS/XRF is a general-purpose absorption spectroscopy beamline at the Synchrotron-light for Experimental Science and Applications in the Middle East (SESAME), Jordan. Herein, its optical layout is presented along with its powerful capabilities in collecting absorption and fluorescence spectra within a wide energy range (4.7–30 keV). The beamline is equipped with a conventional fixed-exit double-crystal monochromator that allows the collection of an X-ray absorption spectrum within a few minutes in step-by-step mode. An on-the-fly scanning mode will be implemented shortly where the acquisition time will be reduced to less than a minute per scan. The full automation of the beamline allows performing successive measurements under different conditions. The different experimental setups and special features available to users are reported. Examples of XRF and XAFS measurements are presented, showing the performance of the beamline under different standard conditions.

## Abstract 2

The XAFS/XRF beamline is the first operational beamline at SESAME and in the Middle East. It is a hard X-ray absorption spectroscopy beamline combining the XRF (X-ray fluorescence) and XAFS (X-ray absorption fine structure) techniques that are applied to a broad scientific community.

## 1. Introduction

The International Center for Synchrotron-light for Experimental Science and Applications in the Middle East (SESAME) is established in Jordan. Its mission is to promote international collaboration in the Middle East and the neighbouring regions using synchrotron light for basic and applied research in physics, chemistry, biology, materials sciences, environmental and medical investigations, archaeological studies and other research areas of relevance to the region. SESAME is a third-generation synchrotron radiation source with a natural emittance of 26 nm rad and a critical energy of 6.05 keV (Vignola *et al.*, 2006 ▸). The facility is composed of a 24 MeV microtron followed by a booster with an energy of 800 MeV that injects into a 2.5 GeV storage ring (Attal *et al.*, 2017 ▸). At present, stored current is 300 mA, which will be optimized to reach the design current of 400 mA in decay mode for a life time of about 24 h.

X-ray absorption spectroscopy (XAS) is an element-selective local probe for assessing the structure and electronic properties of matter in all its atomic structure forms: crystals, glasses/amorphous, liquids and gases (Koningsberger & Prins, 1987 ▸; Stern & Heald, 1983 ▸). This is predominantly a synchrotron technique that is usually not accessible in research laboratories, due to its intrinsic necessity to constantly tune the incoming photon energy, and because it requires a high photon flux. Due to its wide versatility, this technique attracts a large and a growing community of users, comprising environmentalists, electrochemists, biologists, materials and catalysis scientists, *etc*. XAFS/XRF is a beamline dedicated to XAS and it is built in the direction of the tangential fan of the bending magnet at the eighth cell of the SESAME storage ring (D08), allowing access to a wide energy range: from 4.7 to 30 keV, with a high photon flux (2.6 × 10^11^ photons s^−1^ at 10 keV) and a high beam stability to meet the needs of a large number of researchers.

Various fields of research have exploited this instrument since its opening to users (July 2018): environmental science (Morales-Pérez *et al.*, 2021 ▸; El-Hasan *et al.*, 2021 ▸; Shaltout *et al.*, 2021 ▸), energy storage (Khan *et al.*, 2021 ▸; Altin *et al.*, 2021 ▸), materials science (Jamil *et al.*, 2021 ▸; Erat *et al.*, 2021 ▸; Filipponi *et al.*, 2020 ▸; Altaf *et al.*, 2020 ▸; Ozkendir *et al.*, 2019 ▸, 2020 ▸; Bayri *et al.*, 2020 ▸), as well as cultural heritage (Lorentz *et al.*, 2020 ▸) and chemistry and catalysis (Hamo *et al.*, 2021 ▸; Barzgar Vishlaghi *et al.*, 2021 ▸; Amirzhanova *et al.*, 2019 ▸; Bac *et al.*, 2019 ▸; Uzunok *et al.*, 2019 ▸).

In this paper, we report the description of the XAFS/XRF beamline and its potentiality. First of all, we present the beamline layout (from the optical to the experimental hutches), together with the available and the under-development sample setups. We then report on the available acquisition modes, giving information as well on the already ongoing upgrades. Finally, we show examples of spectra obtained at the XAFS/XRF beamline.

## 2. Beamline description

The XAFS/XRF beamline, located at the bending-magnet D08 port, is characterized by one lead-shielded enclosure containing the beamline optics, the so-called optics hutch, and an experimental lead-shielded enclosure used for XAS and XRF experiments.

### 2.1. Beamline layout

The beamline’s outer structure is built up from hutches and cabins. Hutches stand for lead-shielded structures where the synchrotron radiation beam hits optical elements (optics hutch) or the sample (experimental hutch). The full lead shielding of the structure prevents the transmission of radiation to the freely accessible part of the experimental hall. During experiments with X-rays, access to the hutches is prohibited through a Personal Safety System (PSS).

The optics and the experimental hutches were designed on the basis of standard segments and additional elements to fit the total length of the hutches. The roofs have movable slabs aiding in bringing in large equipment and facilitating their installation. A window is installed for each hutch to allow the visual inspection of the equipment while the synchrotron radiation is ‘ON’.

The radiation shielding of the hutches consists of 6 mm of lead for the optics hutch, and 3 mm of lead for the experimental one, where only monochromatic beam can pass in. The experimental hutch is equipped with a so-called user chicane that is linked to the PSS and connected to the beamline control room. This chicane allows users to implement and to control their own experimental setups.

### 2.2. Optical layout

The function of the beamline optics is to deliver a synchrotron radiation beam of the desired characteristics to the experimental end-station. The design goal of the beamline was to find an optimal physical solution to re-use the already existing optics previously installed and operated at the Rossendorf beamline (ROBL), which is a Collaborating Research Group (CRG) beamline at the European Synchrotron Radiation Facility (ESRF) and owned by Helmholtz-Zentrum Dresden-Rossendorf, Germany (Matz *et al.*, 1999 ▸).

**Figure 1:**
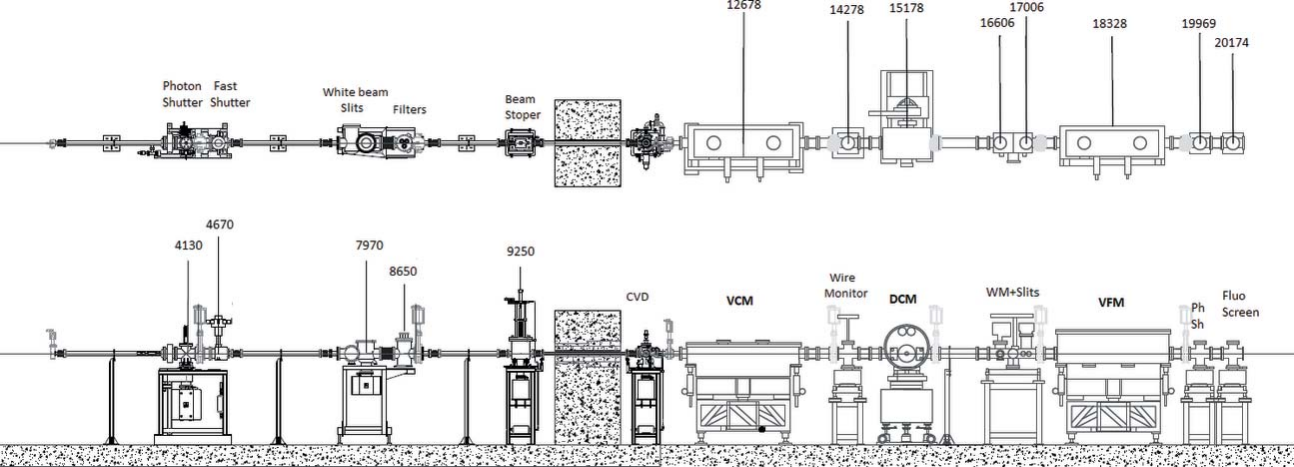
Top and side views of the beamline optics layout showing the different optical components. CVD: chemical-vapour-deposited diamond window; VCM: vertical collimating mirror; WM: wire monitor; DCM: double-crystal monochromator; VFM: vertical focusing mirror; PhSh: photon shutter.

**Figure 2:**
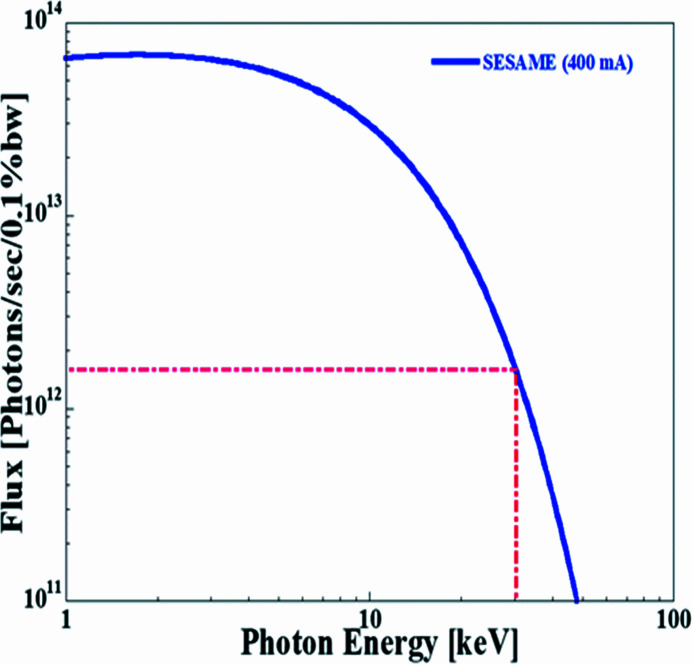
Calculated total flux of the beamline from the bending magnet with 3.2 mrad acceptance in the horizontal and full beam acceptance in the vertical.

The overall layout of the beamline optics is shown in Fig. 1 ▸. The beamline optics implement a fan of 1.9 mrad horizontally of synchrotron radiation from the hard edge of the SESAME bending magnet. The total flux on the sample, positioned at 26 m from the source, as a function of energy, delivered to the beamline under these conditions is shown in Fig. 2 ▸.

The first beamline element, before the photon shutter, is a water-cooled fixed mask located at 4.1 m, which defines the acceptance of the beamline (1.9 mrad horizontally and 0.6 mrad vertically), and reduces the heat load on the optical components by cutting off the off-axis radiation. This mask is followed by a pair of water-cooled white-beam slits located at ∼8 m aiming to define the primary beam size. Next is a water-cooled copper rod equipped with three filters of different thickness implemented for heat management.

The beamline vacuum is separated from the ring vacuum by a CVD window (250 µm thickness). To further protect the storage ring vacuum from ruptures of the beamline vacuum, a fast-closing shutter is installed in the front-end section. Its gauge sensor is placed just before the CVD window in the optical hutch.

The main optical elements are composed of a fixed-exit double-crystal monochromator (DCM) located at ∼15.2 m from the source between a collimating and a cylindrically focusing bent mirror installed before and after the monochromator, and positioned at ∼12.7 m and ∼18.3 m from the source, respectively. The mirrors’ substrates are coated with two parallel stripes of silicon and platinum that are used alternatively (Matz *et al.*, 1999 ▸). The primary water-cooled slits are located at ∼8 m from the source after the diaphragm, also called the fixed mask (4.1 m); they define the horizontal and the vertical dimensions of the polychromatic beam impinging the collimating mirror (VCM, first mirror). Diagnostic tools are installed after each major component, such as beam-position wire monitors that are located after the VCM and the DCM, and an X-ray sensitive screen that is inserted directly after the vertically focusing mirror (VFM, second mirror). A monochromatic shutter is installed at the end of the hutch (∼20.2 m from the source) to allow the user to access the experimental hutch while keeping a constant heat load on the optics.

As reported above, the beamline is designed for the energy range 4.7–30 keV. The lower energy limit is essentially given by the optical and experimental setup, whereas the upper energy limit is imposed by the photon source as shown in Fig. 2 ▸. However, the working energy range allows X-ray absorption fine-structure (XAFS) experiments for almost all chemical elements from Ti onward, since at least one absorption edge is in the operational energy range.

### 2.3. Collimating (VCM) and focusing (VFM) mirrors

Two cylindrical bendable mirrors are installed before and after the monochromator. The first mirror (VCM) is made of a single Si crystal, while the second one (VFM) uses a Zerodur substrate. Both mirrors’ substrates are coated with two parallel stripes of Si and Pt that are used alternatively to achieve a better collimation and focusing of the beam in the vertical dimension. Moreover, the two mirrors, with almost the same grazing-incidence angle, suppress the higher-order harmonics in the monochromatic beam. In addition, the first water-cooled mirror reduces the heat load on the first crystal of the monochromator.

**Figure 3:**
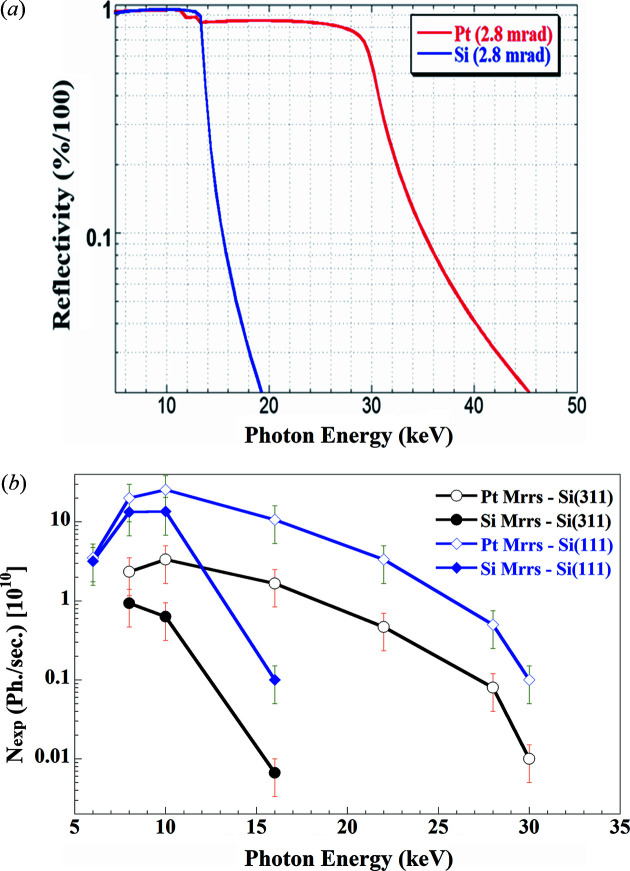
(*a*) Calculated spectral flux reflected by the silicon and platinium coatings of the VCM at a fixed angle of 2.8 mrad. (*b*) Measured flux on the sample [beam size (h × v) = 5 mm × 3 mm] as a function of energy for Si(111) and Si(311) crystals using Pt and/or Si mirror coatings.

The reflective Si and Pt surfaces are 1200 mm long and 70 mm wide, allowing a horizontal acceptance that is widely larger than ∼5.52 mrad on the first collimating mirror. With a fixed angle of incidence of 2.8 mrad and a length of 1200 mm, the first mirror intercepts only ∼0.3 mrad of the vertical beam divergence. The calculated spectral flux for the two coatings is shown in Fig. 3 ▸(*a*), while the measured flux on the sample with a beam size of ∼5 mm × 3 mm (H × V), as a function of energy with different possible combinations of mirror coatings and mono-crystals, is shown in Fig. 3 ▸(*b*). The intensity decrease below 10 keV is due to the absorption of the CVD window.

Both VCM and VFM mirrors are equipped with pneumatic benders. The collimation of the beam is ensured by the first mirror and allows the user to overcome the natural divergence of the beam from the bending-magnet source. This, in turn, improves the energy resolution without limiting the vertical aperture of the primary slits. The VCM is water-cooled and typically gives a maximum transmittance over the complete energy range. The best collimation is obtained by adjusting the mirror bending radius for the given grazing-incidence angle of the mirror in order to reduce the full width at half-maximum (FWHM) of a characteristic sharp structure of a well known standard sample. The second mirror, with an adjustable bending radius, focuses the beam vertically to the experimental end-station, achieving a spatial resolution of 0.1 mm on the sample.

The harmonic suppression is better than 8 × 10^−4^ for all energies when using the silicon stripes. For the platinum stripes it is of the same order of magnitude for energies above 13.5 keV.

### 2.4. Double-crystal monochromator

**Figure 4:**
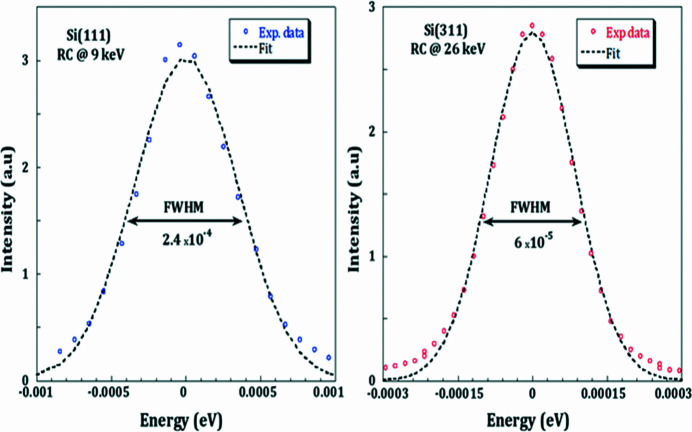
Rocking curve by fine-tuning the DCM second crystal illustrating the energy resolution of the incoming monochromatic beam at FWHM measured with the Si(111) and the Si(311) pairs of crystals at 9 keV and 26 keV, respectively.

A fixed-exit water-cooled double-crystal monochromator (DCM) follows the first mirror (VCM). The DCM is a donation from Diamond Light Source originally installed at the I11 beamline (Thompson *et al.*, 2009 ▸; Tang *et al.*, 2007 ▸), and it replaced the originally installed monochromator donated by the ROBL facility which has obsolete and complicated mechanics. The newly installed DCM is equipped with slots for two pairs of crystals that can be exchanged by an in-vacuum translation. The Si(111) pair of crystals was delivered with the donated DCM, while the Si(311) was manufactured by the optics group of Argonne National Laboratory and donated to SESAME. Both pairs are installed and cover a photon energy range from 4.7 to 30 keV. The fixed exit is maintained by translating the second crystal vertically in combination with a ∼180 mm-long crystal. The combination of a collimating mirror with the DCM ensures an energy resolution that is almost matching that of the crystals: Δ*E*/*E* ≃ 2.4 × 10^−4^ for Si(111) at energy 9 keV and 0.6 × 10^−4^ for Si(311) at energy 26 keV (Fig. 4 ▸). The goniometer’s centre of rotation lies on the centre of the beam and is adjusted manually in height during the installation. The surfaces of both first crystals, Si(111) and Si(311), lie within an accuracy of ±0.1 mm in the centre of rotation. The pitch and roll movements of the second crystal are controlled by a stepper motor for coarse adjustments, and a piezo motor for fine adjustments. The roll movement is controlled by a stepper motor on the first crystal.

The DCM can be either moved in step-scanning mode that ensures a high beam position stability, or continuously allowing for faster scans (to be implemented). Conventional quick-XAFS, or alternatively ‘continuous’ XAFS, scans can be performed by moving the DCM continuously through the energy range (Bragg angle) of interest, while the encoder readout of the Bragg angle and the detectors are sampled simultaneously (Frahm, 1988 ▸, 1989 ▸). With conventional quick-scanning mode, one typically obtains a time resolution in the range of a few seconds for the X-ray absorption near-edge structure (XANES) region and up to a few minutes for the extended X-ray absorption fine-structure (EXAFS) region. In such a short period, it is difficult to set the second crystal in the exact position for a fixed-exit beam and thus a so-called pseudo channel-cut mode of the DCM will be used resulting in a vertical beam movement, over a 500 eV scan, of less than ∼0.1 mm at lower energies, and less than ∼0.6 mm for energies above 14 keV.

Both first and second crystals of the DCM are indirectly water-cooled, and controlled by a chiller (±0.01 K). The crystals are mounted over an indium foil onto a copper block. Downstream of the monochromator a water-cooled copper block allows passage of only the monochromatic beam (offset of 18 mm).

### 2.5. Other optical elements

In addition to the mirrors and the monochromator, the optics hutch contains various units of slits, filters, beam-position monitors and a retractable sensitive screen. The slits unit is composed of a couple of vertical and horizontal independent tungsten carbide knives that move with an accuracy of 10 µm. The filter unit has six absorber foils to attenuate the white beam. The beam-position monitors consist of scanning tungsten wires. The motions of all optical components are motorized, mostly with stepper motors, and controlled by a Linux Scientific (LS) workstation-based system. The EPICS control system is used to control the optics (mainly the motion system), via a Galil controller, and the experimental part (signal from detectors, high voltage, motors, *etc*.). The control system also includes interlock components for the vacuum, beam shutters and the cooling of components exposed to the white beam in a separate control system called the Equipment Protection System (EPS). Moreover, the Personal Safety System (PSS) ensures access to the hutches during the operation of the beamline.

### 2.6. Endstation

The experimental hutch is designed for monochromatic beam operation only. As mentioned in Section 2.1, a water-cooled copper block located after the monochromator allows for the passage of monochromatic beam only. Both the experimental and the optics hutches are equipped with a crane allowing 1000 kg load capacity. A movable optical table (six axes of freedom) is mounted allowing the alignment of any sample environment. Located at 26 m from the source, the heavy duty base model of the sample stage consists of four motors to centre the sample and the sample holder to the beam: *z*, rotation, *x* and *y*. These have a maximum load capacity of 10 kg. Small top modules (rotation, swivel, *x*, *y*) can additionally be used to orientate the sample with respect to the beam. A sample holder is available for solid/reference samples. All stages have micrometre resolution. To optimally condition the beam, various cameras are available to determine the beam position, shape and movement.

A flexible data acquisition Python-based software is currently available to collect XAFS data in different modes allowing the selection of different energy regions with different scanning steps, as well as setting different acquisition times per point. An EPICS-based GUI interface is under examination and will be available soon which will allow an easy control over step or continuous scanning mode (once implemented). The data of the spectra are in ASCII format for XAFS and 1D XRF, which can be read by almost any conventional data analysis program.

### 2.7. Detectors

Samples can be measured in both transmission and fluorescence geometries. Available detectors include two 15 cm-long and one 30 cm-long low-noise ion chambers from Ohyo Koken Kogyo Co. Ltd, Japan. A simple gas-mixing system is available to fill the ion chambers with the appropriate mixtures of inert gases. The ionization chambers and the sample manipulator are on a rail system, allowing a modular setup to the beam. Low-noise current pre-amplifiers (SR750, Standford Research Systems) are located directly below the ionization chambers and are remotely controlled. Fluorescence yield can be measured with 64 silicon drift detectors (SDDs) (Rachevski *et al.*, 2019 ▸). The 64-array detector is a completely depleted volume of *n*-type 450 µm-thick silicon wafer with eight monolithic arrays. The total sensitive area of the matrix is 499  mm^2^. The energy resolution of a single square cell is 150 eV FWHM at 5.9 keV. The 64-array detector runs with its intrinsic built-in pulse-processing electronics and allows higher counts with low dead-time which results in an increase of the detector efficiency. A spare single-element SDD (AXAS-D) can also be used. The AXAS-D detector by KETEK is a digital system, with a 40 mm^2^ SDD chip collimated to 30 mm^2^ active area. The single-element AXAS-D solid-state detector is combined with digital pulse processing electronics (XIA-Mercury), and can be utilized with different peaking times allowing the choice/compromise between high count rates or high energy resolution (∼150 eV).

## 3. Beamline performance

**Figure 5:**
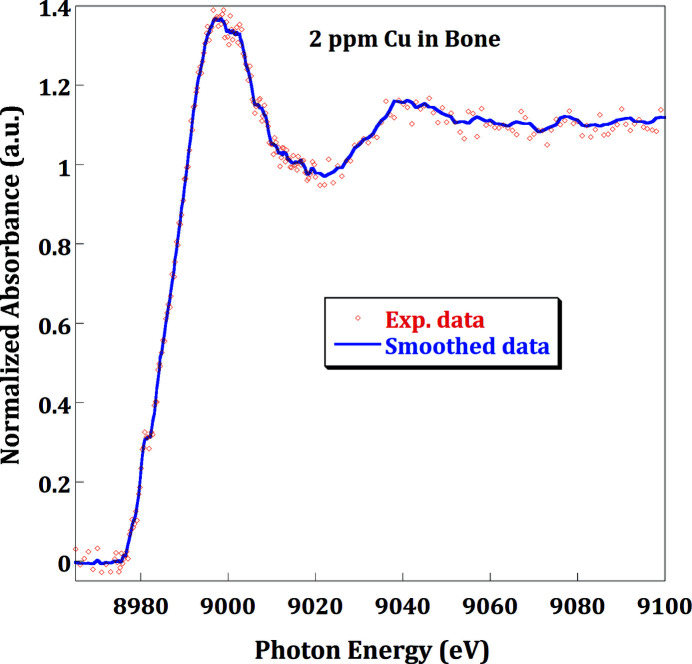
Cu *K*-edge XANES spectrum averaged over 12 repetitive scans collected using the INFN 64-SDDs detector on an archaeological sample (bone) with only 2 p.p.m. Cu concentration. The experimental data (pink dots) were smoothed using a three-point-smoothing attempt with three repetitions (blue)

For XAFS/XRF, spectra can be acquired in step-by-step mode (continuous mode will be implemented shortly). In this acquiring mode, the acquisition is performed in a static mode, as soon as all the DCM motors have reached the desired positions. Since the dead-time due to the motor movements in step mode is less than 2 s per point, an XAFS scan is performed in a few tens of minutes. In transmission mode, at least three repeats should be foreseen to check for reproducibility. In fluorescence mode, at least 3–15 repeats are necessary to access a good signal-to-noise ratio in the EXAFS (XANES) region, depending on the sample. As a result, working in fluorescence mode is, currently, very time-consuming. Moreover, in fluorescence mode, measuring the emission lines of highly diluted elements is the most common type of experiment, and the signal coming from the single-channel detector appears to be not reasonably sufficient to access less than a few tens of p.p.m. of concentration. To increase the efficiency and the sensitivity of the beamline fluorescence experiments, a 64-channel SDD has been developed in collaboration with INFN (Italy), Elettra (Italy) and SESAME (Rachevski *et al.*, 2019 ▸). The detector has been recently installed at the XAFS/XRF beamline and its performances have been verified. With this multi-channel SDD, it was possible to collect decent XANES data at the Cu *K*-edge, in archaeological materials with a very low (∼2 p.p.m.) Cu content (Fig. 5 ▸). Due to the low Cu concentration (measured by XRF) in the archaeological sample, XANES were collected by scanning the energy in the step-by-step mode. Different energy ranges of the spectrum were used with a 0.5 eV step at the pre-edge and white-line region. The integration time for each point is variable for each energy range with 20 s per point at the critical energy part (near the absorption edge). A total of 17 XANES spectra acquired in 50 min per spectrum were collected and averaged for the decent spectrum shown in Fig. 5 ▸.

**Figure 6:**
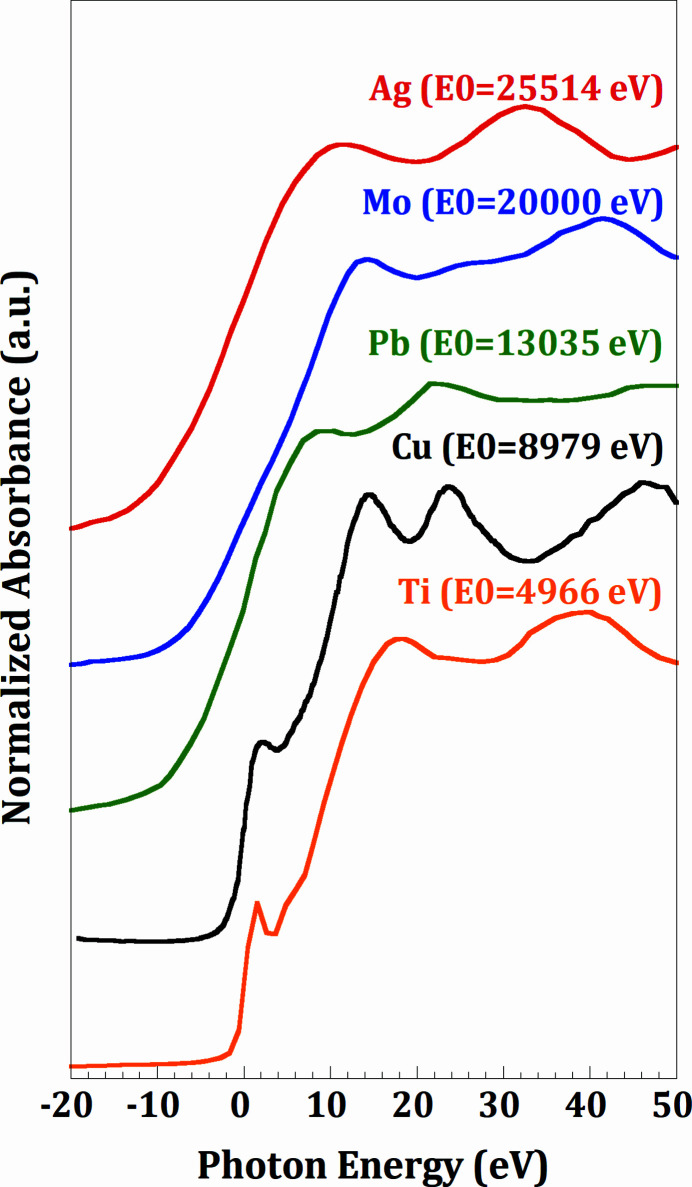
Normalized XANES from reference metal foils collected at different energies with Si(111) crystal for the Ti and Cu *K*-edges (4966 eV and 8979 eV, respectively) and Pb *L*-edge (13035 eV), and with Si(311) crystal for the Mo and Ag *K*-edges (20000 eV and 25514 eV, respectively).

**Figure 7:**
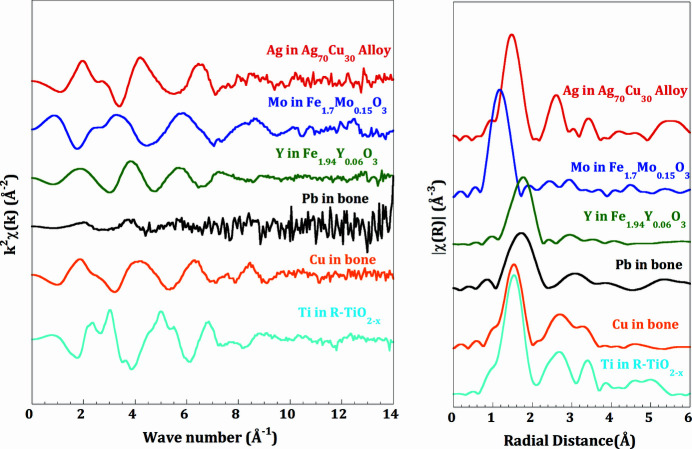
*k* ^2^-weighted EXAFS spectra (left) and their corresponding Fourier transform magnitude (right) collected at different edges covering the full energy range of the beamline. Samples are from different research projects conducted at the XAFS/XRF beamline: Ag *K*-edge (25.514 keV) in AgCu alloys as electro-catalyst for oxygen reduction reaction; Mo *K*-edge (20.000 keV) in Fe_1.7_Mo_0.15_O_3_ for nonstoichiometric systems for ionic conductivity behaviour; Y *K*-edge (17.038 keV) in Fe_1.94_Y_0.06_O_3_ for nonstoichiometric systems for ionic conductivity behaviour; Pb *L*_III_-edge (13.035 keV) in ancient human bone (3000 BC); Cu *K*-edge (8.979 keV) in ancient human bone (3000 BC); Ti *K*-edge (4.966 keV) in R-TiO_2–*x*_ samples for visible-light-driven photocatalysis.

In Fig. 6 ▸, XANES spectra are reported for reference metal foils for the *K*-edges of Ti (4.966 keV), Cu (8.979 keV) and the *L*_III_-edge of Pb (13.035 keV) collected with the Si(111) crystal, in addition to the Mo (20.0 keV) and Ag (25.514 keV) *K*-edges collected with the Si(311) crystal. Fig. 7 ▸ shows different examples of EXAFS spectra and their corresponding Fourier transforms, collected on different samples from different research domains, namely AgCu alloys as electro-catalyst for the oxygen reduction reaction (Ag *K*-edge), Fe_1.7_Mo_0.15_O_3_ and Fe_1.94_Y_0.06_O_3_ for nonstoichiometric systems for ionic conductivity behaviour (Mo and Y *K*-edges, respectively), Pb and Cu in ancient bone from 3000 BC, with concentrations of 116 p.p.m. and 101 p.p.m., respectively, and R-TiO_2–*x*_ sample for visible-light-driven photocatalysis (Ti *K*-edge). All EXAFS data were collected in fluorescence mode except for the Ag and Ti edges. More examples of results collected at the XAFS/XRF beamline are reported in the literature (Bac *et al.*, 2019 ▸).

## 4. Conclusions

The XAFS/XRF beamline at SESAME started its operational phase in 2018. In summary, the optical layout and experimental setups available at the XAFS/XRF beamline at SESAME have been described in this paper along with a few examples of X-ray absorption spectra collected under the different standard conditions at different energies. The beamline capabilities and performances were highlighted. The scientific results published thus far demonstrate the unique possibilities of the XAFS/XRF beamline at SESAME. These publications cover a wide range of research fields in materials sciences, catalysis, environmental science and geosciences.
